# Pediatric SARS-CoV-2 Seroprevalence, Oregon, USA, November 1, 2020–June 30, 2022

**DOI:** 10.3201/eid2908.230471

**Published:** 2023-08

**Authors:** Rebecca A. Falender, Paul G. Mitchell, Judith A. Guzman-Cottrill, Paul R. Cieslak, Melissa Sutton

**Affiliations:** Oregon Health Authority, Portland, Oregon, USA (R.A. Falender, P.R. Cieslak, M. Sutton);; Oregon Health and Science University School of Medicine, Portland (P.G. Mitchell, J.A. Guzman-Cottrill)

## Abstract

We estimated SARS-CoV-2 seroprevalence in children in Oregon, USA, at 6 time points. Seroprevalence increased linearly during November 2020–December 2021 and peaked in February 2022 at 38.8% (95% CI 32.8%–46.5%). We observed no increase in the seroprevalence trend after widespread school reopening. Seroprevalence estimates complement case-based cumulative incidence.

Through June 30, 2022, a total of 140,820 pediatric cases of COVID-19 had been reported in Oregon, USA, representing ≈17.3% of all reported COVID-19 cases in the state. However, understanding the true burden of pediatric COVID-19 infection poses a challenge. Children are more likely to have asymptomatic or mild disease, and pediatric infections are, therefore, less likely to be reported to public health authorities (*1*,*2*). Clarifying pediatric SARS-CoV-2 prevalence is important because it is well established that children can transmit SARS-CoV-2 to other children and adults (*3*,*4*). In addition, children are at risk for severe complications, including postinfectious multisystem inflammatory syndrome in children (MIS-C) (*5*). Seroprevalence provides additional insight into the true cumulative incidence of COVID-19 in children.

## The Study

To estimate the seroprevalence of COVID-19 infection in children in Oregon, blood was collected in 6 phases during November 1, 2020–June 30, 2022, from a cross-sectional convenience sample and tested for SARS-CoV-2 nucleocapsid IgG, in alignment with the World Health Organization seroepidemiologic investigation protocol (*6*). We recruited Oregon healthcare facilities with >6 inpatient pediatric hospital beds to participate in this study and asked them to provide <100 specimens per phase; 5 facilities agreed to participate. We asked facilities to submit random samples of deidentified residual serum samples from patients <17 years of age visiting any ambulatory, emergency, or inpatient healthcare setting and to include specimen collection date and patient’s date of birth. The initial round of sampling was November 1–December 31, 2020. We extended the project timeline and collected additional samples bimonthly during October 1, 2021–June 30, 2022.

Specimens were stored according to instructions provided by the test manufacturer and transported to the Oregon State Public Health Laboratory (Hillsboro, Oregon, USA). We tested the specimens with a SARS-CoV-2 IgG assay (Abbott Laboratories, https://www.abbott.com), which detects antibodies to the nucleocapsid protein of SARS-CoV-2. Nucleocapsid IgG immunoassays detect antibodies produced after infection and do not detect antibodies produced after vaccination with vaccines licensed for use in the United States. The manufacturer reports test sensitivity (Sn) of 100% (95% CI 95.9%–100%) at >14 days past symptom onset and specificity (Sp) of 99.6% (95% CI 99.1%–99.9%). We calculated unadjusted seroprevalence estimates for each collection period as the percentage of all specimens that tested positive. We adjusted seroprevalence estimates for test performance as observed prevalence + (specificity – 1) divided by sensitivity + (specificity – 1) (*7*). 

We obtained adjusted 95% CI with parametric bootstrapping (*8*). Because SARS-CoV-2 antibody detection is dependent on test timing and assay, we also performed sensitivity analysis with seroprevalence estimates adjusted for declining assay sensitivity over 130 days of convalescence (*9*,*10*) (Appendix) We used R version 4.1.2 (The R Foundation for Statistical Computing, https://www.r-project.org) for all analyses.

We collected 1,869 specimens from 5 facilities during 6 phases. The mean number of specimens collected during each phase was 312 (range 215–438). Overall, we observed a strong linear trend (p = 0.001) for adjusted seroprevalence estimates during November 1, 2020—December 31, 2021; seroprevalence increased by ≈0.7% per 4-week period during that period (Figure). After the Omicron surge, adjusted seroprevalence increased sharply from 13.4% (95% CI 9.8%–18.4%) in December 2021 to 38.8% (95% CI 32.8%–46.5%) in February 2022. Adjusted seroprevalence estimates then decreased but did not return to pre–February 2022 levels (Table). Adjusting for declining assay sensitivity over time led to estimates that were larger and less stable over convalescence (Appendix).

**Figure:**
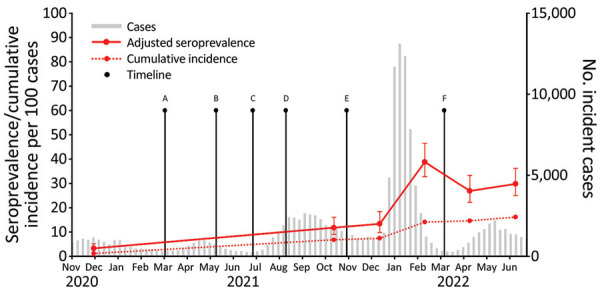
Adjusted SARS-CoV-2 nucleocapsid seroprevalence, incident cases, and cumulative incidence in children, Oregon, USA, November 1, 2020–June 30, 2022. We calculated cumulative incidence estimates at the midpoint of each collection period and plotted adjusted seroprevalence estimates with 95% CIs (error bars). Incident cases and cumulative incidence are plotted by epidemiologic week. Points A through F denote key timepoints: A, all public schools required to offer in-person instruction for grades K–5 by March 29, 2021, and grades 6–12 by April 19, 2021; B, COVID-19 vaccine available for children 12–15 years of age on May 12, 2021; C, statewide mask mandates lifted on June 30, 2021; D, statewide mask mandates reinstated on Aug 13, 2021; E, COVID-19 vaccine available for children 5–11 years of age on November 2, 2021; F, statewide mask mandates were lifted on March 12, 2022. Incident cases and case-based cumulative incidence estimates were calculated at the midpoint of each collection period and are obtained from pediatric COVID-19 cases reported to Oregon Health Authority since the beginning of the pandemic.

**Table:** Seroprevalence of SARS-CoV-2 nucleocapsid antibodies in children, Oregon, USA, November 1, 2020–June 30, 2022*

Collection dates	Sample size	Unadjusted seroprevalence (95% CI)	Adjusted seroprevalence (95% CI)
2020 Nov 1—Dec 31	438	0.032 (0.015–0.048)	0.033 (0.002–0.052)
2021 Oct 1—Oct 3	370	0.122 (0.090–0.159)	0.118 (0.090–0.161)
2021 Dec 1—Dec 31	278	0.137 (0.099–0.183)	0.134 (0.098–0.184)
2022 Feb 1—Feb 28	215	0.391 (0.325–0.459)	0.388 (0.328–0.465)
2022 Apr 1—Apr 30	279	0.272 (0.221–0.329)	0.269 (0.222–0.333)
2022 Jun 1—Jun 30	289	0.301 (0.249–0.358)	0.298 (0.250–0.362)

*We calculated adjusted prevalence as observed prevalence + (specificity – 1) divided by sensitivity + (specificity – 1). Adjusted 95% CI obtained with parametric bootstrapping.

## Conclusions

Our repeated cross-sectional study estimated pediatric seroprevalence in Oregon at 6 points across 20 months of pandemic response. During that period, K–12 public schools reopened statewide, vaccines were rolled out in 2 phases to children 12–15 and 5–11 years of age, and universal indoor masking mandates remained in place during the school year until March 12, 2022 (Figure). After widespread school reopening in early 2021, with a masking mandate in place, pediatric seroprevalence in October 2021 was 11.8%, and no increase in trend was observed. The only sudden increase in pediatric seroprevalence followed the Omicron surge in early 2022. Seroprevalence began to decline after its February 2022 peak but did not return to pre-Omicron levels.

Seroprevalence can provide more accurate estimates of the true cumulative incidence of SARS-CoV-2 infection than case reporting to public health entities does, because seroprevalence data capture evidence of previous infection in persons who are not tested through the traditional healthcare system because of asymptomatic or mild disease, lack of testing access, refusal to test, or self-testing at home (*11*). We estimated 1.7–2.8 times the number of infections in children from seroprevalence than the reported cumulative incidence in Oregon (Figure). This total is a lower degree of underascertainment than had been reported in seroprevalence studies of children during May–July 2021 in the United States (*1*).

Because seroprevalence studies measure circulating antibodies at the time of testing and SARS-CoV-2 antibodies wane over time, seroprevalence is limited in its ability to estimate cumulative incidence as the pandemic progresses (*12*). In addition, time to seroreversion is dependent on the target antigen and the assay used (*9*,*10*,*13*). One study found a mean time of seroreversion of 19 weeks with use of the Abbott IgG immunoassay, compared with 91 weeks using the Roche pan-Ig immunoassay (*13*). In our study, waning immunity was apparent as estimated seroprevalence decreased following its Omicron-related peak in February 2022. A sensitivity analysis, adjusting for declining sensitivity of the Abbott immunoassay, partially accounted for this decline; more complex models have been published to correct seroprevalence estimates for assay performance (*13*). However, now that essentially all persons in the United States have been infected with SARS-CoV-2, the use of an assay with waning sensitivity to remote infections may permit continued examination of more granular temporal changes in seroprevalence in the context of changing policy and variant predominance.

The Centers for Disease Control and Prevention began collecting pediatric seroprevalence data for the Multistate Assessment of SARS-CoV-2 Seroprevalence in Commercial Labs (MASS-C) in July 2020 (*14*). MASS-C estimates of pediatric seroprevalence in Oregon are consistently higher than our estimates (*15*). Although MASS-C similarly derives its estimates from a convenience sample of serum specimens, testing is performed with the Roche antinucleocapsid total antibody assay (*14*). In addition, MASS-C estimates are weighted for age and sex, and the demographics of that pediatric population may demographically differ from our study population.

We obtained our convenience sample from children who received care from large pediatric healthcare facilities throughout the state; findings are not necessarily generalizable to the entire state pediatric population. Limitations of seroprevalence testing include lack of antibody development by some infected persons (including immunocompromised persons) and, in others, waning of antibodies to undetectable levels, such that seroprevalence becomes a less reliable proxy for cumulative incidence as the duration of the pandemic increases (*12*).

Traditional public health case-based reporting substantially underestimates the burden of COVID-19. In this study, seroprevalence estimates made using an assay with waning sensitivity to remote infections provided evidence that the widespread reopening of schools with a masking mandate in place did not increase the rate of pediatric SARS-CoV-2 infections. Case-based cumulative incidence estimates failed to capture the magnitude of the Omicron variant’s effect on Oregon’s pediatric population. Serosurveillance of SARS-CoV-2 antibodies in Oregon’s pediatric population complements case-based surveillance and can inform future public health interventions and policy decisions.

Appendix Additional information about pediatric SARS-CoV-2 seroprevalence, Oregon, USA, November 1, 2020—June 30, 2022.
