# Supplementary material for: Pediatric SARS-CoV-2 Seroprevalence, Oregon, USA, November 1, 2020–June 30, 2022
Source: Emerg Infect Dis. 2023 Aug;29(8):1672–5. doi: 10.3201/eid2908.230471 (PMC10370852; doi:10.3201/eid2908.230471)
Supplement: Appendix — Additional information about pediatric SARS-CoV-2 seroprevalence, Oregon, USA, November 1, 2020—June 30, 2022. [file 23-0471-Techapp-s1.pdf]

Article DOI: http://doi.org/10.3201/eid2908.230471

EID cannot ensure accessibility for supplementary materials supplied by authors. Readers who have difficulty accessing supplementary content should contact the authors for assistance.

Pediatric SARS-CoV-2 Seroprevalence, Oregon, USA, November 1, 2020–June 30, 2022

Appendix

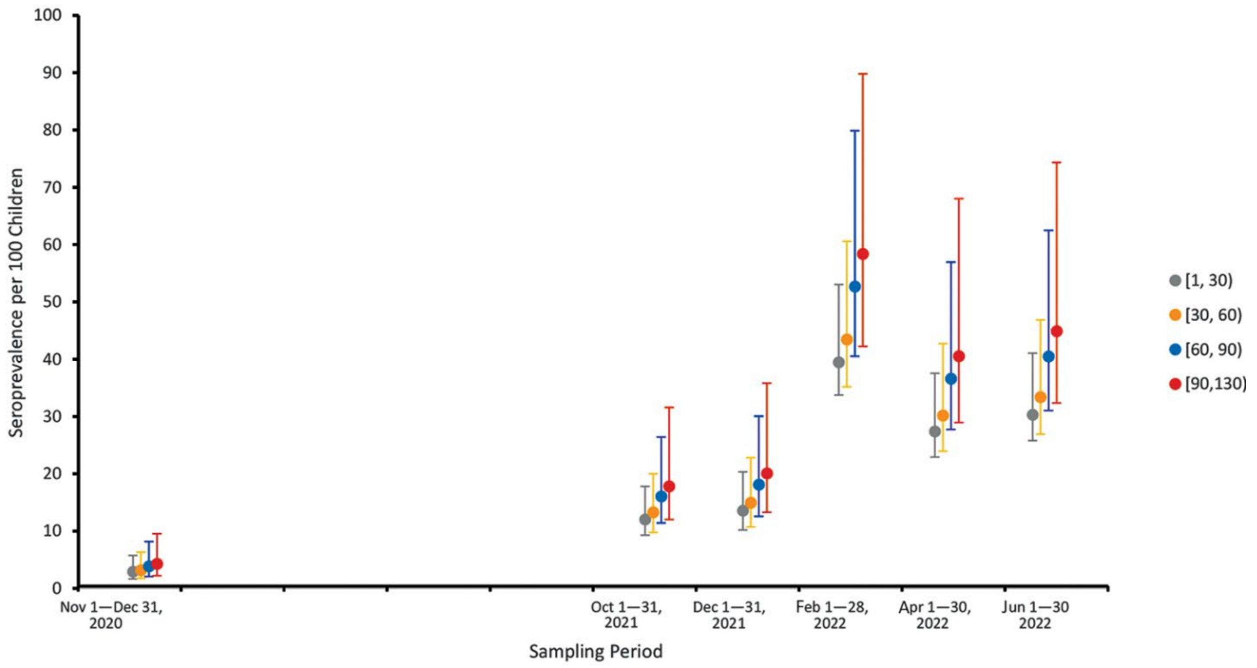

Appendix Figure. Sensitivity analyses with estimates adjusted for test performance at 4 time periods over 130 days of convalescence using the formula observed prevalence + (specificity – 1) divided by sensitivity + (specificity – 1). Sensitivity estimates of IgG nucleocapsid assay at 4 time periods over convalescence were obtained from previous assay performance analyses (*1*,*2*). The manufacturer's reported specificity of 99.6% (95% CI 99.1%–99.9%) was used for all adjustments. Adjusted 95% CI were obtained with parametric bootstrapping.

References

1. Peluso MJ, Takahashi S, Hakim J, Kelly JD, Torres L, Iyer NS, et al. SARS-CoV-2 antibody magnitude and detectability are driven by disease severity, timing, and assay. Sci Adv. 2021;7:eabh3409. PubMed https://doi.org/10.1126/sciadv.abh3409

2. Stone M, Grebe E, Sulaeman H, Di Germanio C, Dave H, Kelly K, et al. Evaluation of commercially available high-throughput SARS-CoV-2 serologic assays for serosurveillance and related applications. Emerg Infect Dis. 2022;28:672–83. PubMed https://doi.org/10.3201/eid2803.211885
